# A biased ADHD discourse ignores human uniqueness

**DOI:** 10.1080/17482631.2017.1319584

**Published:** 2017-05-22

**Authors:** S. I. Erlandsson, E. Punzi

**Affiliations:** ^a^ Department of Social and Behavioral Science, University West, Sweden; ^b^ Department of Psychology, Gothenburg University, Sweden

There is a tendency in our current society to perceive human experiences, emotions, behaviors, relations, and difficulties as expressions of neurobiological functions and dysfunctions. Studies indicating that hardships like those just described should be understood as representing biochemical or neuroanatomical deviations are published in academic journals and presented in popular media. Although these studies represent a rather one-sided research focus, they create a sense that we live in an era where scientific progress is greater than ever before, with no need to look backwards (Lewis, 2006). This special issue in IJQHW on “Understanding children and young adults diagnosed with ADHD—a critical standpoint” lends its voice to experienced clinicians and researchers coming from a diversity of disciplines and countries in the world. Included here are papers written by researchers who criticize the current situation, but also point to alternative understandings, approaches, ethics, and treatments. Epigenetic research can teach us that we must pay attention to the impact of the social context on brain development. This impact includes early-life experiences but also how epigenetic pathways responsible for detecting the input from the environment act, and what influences phenotypic variations have across generations. As Champagne (2013) summarizes in a study explaining the contribution of epigenetic factors to our present knowledge of the link between the human brain and the environment: “Scientific progress is made through the careful research steps that build a foundation that is larger than the sum of its parts” (p. 634). We simply need to discover more about the nature-nurture interaction to understand how to meet the challenges that we have to face in a fast changing society.

To disregard individuals’ experiences, even from an early age, and the influence on emotional and cognitive functions those experiences may have, is one way to reduce humans to neurobiological objects. In times when, more than ever, we need to acknowledge and understand the diversity of peoples’ experiences, the current focus on diagnostics and brain dysfunctions in people instead contributes to the marginalization and stigmatization of individuals. An important side of the discourse on ADHD relates to academic study books and how the authors in these books describe underlying mechanisms, and more specifically the genetics, of ADHD to students who will become future healthcare professionals. In one of the articles in this special issue Meerman, Batstra, Hoeckstra, and Grietens (2017) examined a section on ADHD in study books used for (pre)master programs from 10 universities in the Netherlands. They applied a framework including four categories (A,B,C,D) for defining how the authors of the books mention or omit effect sizes of quantitative and molecular ADHD genetics. The presence of effect sizes related to quantitative genetics such as twin studies and molecular genetics such as candidate gene studies were scored. Approximately half of the selected books did not mention “the low explained variance of molecular genetic studies”. About one quarter mentioned both quantitative and molecular effect sizes; however, in the final quarter of study books, no effect sizes were mentioned. The authors note that an important gene related to ADHD might have been around for 50,000 years, and note that it has not been clarified why the expression of this gene suddenly should provoke certain problematic behaviors in children.

In line with the idea of scientific progress, bio-psychiatry became dominant over more humanistic traditions during the late-twentieth century. ADHD is a paramount example of our society’s tendency to emphasize neurobiological explanations for human behavior, emotion, and reactions. A precursor of ADHD was included in the *DSM II* in 1968, namely Hyperkinetic Reaction of Childhood. In later editions of DSM, the terms Attention Deficit and Attention Deficit Hyperactivity Disorder were included. In *DSM-5*, ADHD is presented as a neurodevelopmental disorder. This means that the highly elastic criteria for the diagnosis, *a priori* become viewed not only as a disorder but also as a disorder within the nervous system. National authorities such as The National Institute on Mental Health in the USA and The National Board of Health and Welfare in Sweden present restlessness, inattention, and so called hyperactivity among children as signs of neurodevelopmental dysfunctions, and legitimize this perspective with reference to contemporary research findings. Moreover, health professionals sometimes proclaim themselves as experts on children’s problems overall, and as experts they may defend their conviction also in the media, as in the following example. The following quotation is about the reason why troubled children stay out of school for long periods of time (translated to English, DN, 2017):

Still there are many myths about these children’s behavior. Such as that people believe it is about incompetent parents or that the behavior is due to moral character and will disappear if the child is adequately nurtured. People look for psychological explanations. These attitudes hamper understanding of what the real causes are.

Noteworthy here is that “real causes” mentioned by the interviewed health professional neither refer to as a child’s emotional experiences nor to the social circumstances of that child, for example care and potential parental hardships. How can we understand what real causes are if we exclude human experience and the influence of those experiences on our behavior over time? Who dares to claim to know the real causes of all children’s dysfunctional behaviors? These kinds of “myths” declared by professionals and experts in the media must be met and discussed within the scientific community by scholars who cherish an interdisciplinary approach to human burdens.

The media has a significant role in spreading research findings all over the world. Ponnou and Gonon (in press) identified 159 articles in French newspaper giving facts and opinions on ADHD over a period of 20 years (1995–2015). They compared these results with how previous studies covering French TV programs and specialized press, aimed at social workers, portrayed ADHD. Interestingly, the authors found that the biological arguments had become less frequent during the most recent years. Out of 159 articles, only 11 claimed that school failure could be solved by medication. Most French newspapers recognize ADHD as a real syndrome that may require medication in combination with other therapeutic approaches, but not as a neurological disease that can be treated by a drug. In French TV programs, however, ADHD was described as an inherited neurological disease and medication was frequently presented as offering protection against school failure. The information presented in TV programs is rather alarming, especially as the authors conclude that television is the main source of Europeans’ health information. In another part of the world, Harwood, Jones, and Bonney (2017) explored how the Australian newsprint media use metaphors for the description of ADHD, medicalization, and behavior. The authors emphasize that metaphor usage by the media is important to investigate since metaphors are influential in communicating ideology. For example they found that so-called “scientific breakthrough metaphors” provided a convincing narrative of ADHD in 123 of 453 articles. Metaphors that were used in the media allowed “an easy passage” of behavior into unquestioned medicalized fields. Based on their findings the researchers concluded that the media provides an unobjectionable view of the medicalization of child behavior. As medical prescriptions for ADHD are found to be disproportionally high in socioeconomic disadvantaged areas (Harwood, 2010; Watson, Arcona, Antonuccio, & Healy, 2014), a broader discussion in the media on the medicalization and the diagnosis of ADHD should be encouraged (Gonon, Konsman, Cohen, & Boraud, 2012; Winter, Moncrieff, & Speed, 2015).

One area that has attracted academic attention is the tendency to perceive schoolteachers as being in the position of identifying children who should be diagnosed with ADHD. This means that teachers become an extension of the medical profession. For example in special education textbooks children diagnosed with ADHD are presented as dysfunctional and the need for alternative perspectives is acknowledged (Freedman, 2016). Meerman, Batstra, Grietens, and Frances (2017) contribute an article that provides alternatives that encourage educational institutions and individual teachers to rely on their professional skills and facilities, and to be cautious about explaining challenging behaviors as signs of pathology. The teachers could instead reflect on how children are expected to behave. Accordingly, the authors provide an overview of research. This overview, for example, concerns the importance of maturity and birth month when approaching children with challenging behaviors. They also discuss the noticeably weak research findings concerning ADHD as a brain dysfunction, as well as concerning the long-term benefits of medication.

The time has come to ask ourselves how the tendency to portray children as dysfunctional has evolved. Researchers have acknowledged the influence from the pharmacological industry. Other researchers have investigated the approach toward and perception of children, for example Fass (2016), who describes a current tendency to perceive children as the target of parental control. If the child is perceived as deviant, the blame is on the parents and they might become flooded with advice. According to Fass there is increasing parental disappointment with completely normal children who do not live up to expectations of control and perfection, and thus become perceived as dysfunctional. The increasing tendency to perceive children as dysfunctional could thus reflect a striving for the “desired life-world”. Such a striving also surfaces in school settings in which children who for some reason have difficulties adjusting to expectations and demands run the risk of becoming diagnosed as inherently dysfunctional (Nilsson Sjöberg, 2016). Thereby, contextual factors, such as socioeconomic marginalization and/or exclusion and even bullying, become neglected. However, there is another reason for the increasing rate of children diagnosed with ADHD; the tendency to diagnose children exposed to marginalization and low socioeconomic conditions with ADHD. These children live in a world that is far from idealized and a diagnosis might thus conceal social as well as interpersonal difficulties and traumas.

In the relatively short history of psychiatry and mental health care, there are numerous examples of dubious diagnoses, interventions and explanations of human suffering, of which hysteria is an obvious example. Bio-psychiatric explanations of this obscure entity pointed to the uterus being dysfunctional or ill-positioned, and treatment could involve isolation, electrotherapy, genital massage, and other manipulations of the body (Brouselle et al., 2014; Latham, 2015). Other failures include the view of sexually active women as deviant and pathological (Johannisson, 2015), and the definition of homosexuality as a medical pathology (APA, 1968). Treatment interventions have often been degrading and even dangerous. Lobotomy, a highly dangerous operation, is now condemned, but during the 1940s and 1950s was recommended and presented as a miracle cure in the popular media (Johnson, 2009). According to Ögren, Sjöström, and Bengtsson (2000) around 4500 patients with various types of psychiatric illness were lobotomized in Sweden, and the mortality of those who were operated upon during 1947–1955 was 7.4%. Some of them were children with intellectual disabilities. Sigrid Hjertén, born 1885, and one of our precious Swedish expressionist painters, died after lobotomy executed at Beckomberga hospital in Stockholm 1948. The Swedish National Board of Health and Welfare was at the time informed about the high mortality rate among those who were lobotomized, but these facts did not lead to any official precautions being taken (Ögren, 2007). During the 1950s, thalidomide was presented as a safe, completely non-poisonous medicine and pregnant women were prescribed it to reduce sleeping problems and morning sickness, with the result that at least 10,000 children were born with severe dysfunctions, and a considerable proportion of them died within weeks after birth (Klausen & Parle, 2015).

It is easy to judge researchers and clinicians who acted according to the beliefs and ideals of their time. Considering the historical retrospect, how will future generations analyze, explain, and criticize the way children’s behaviors today are classified and diagnosed as expressions of neurobiological dysfunctions? Trying to predict future criticisms might help us to act and provide alternatives. A beginning is to learn through children’s own stories and representations how they perceive their troubles and what they themselves experience as obstacles to adaptation and wellbeing. We often proclaim that the time has come to listen to what children have to say, but this is far from being realized. One of the papers on this special issue brings to the forefront theoretical and clinical perspectives illustrating how psychoanalytical psychotherapy can be of use for understanding and caring for a child diagnosed with “DAMP” (Deficits in Attention, Motor control, and Perception), a diagnosis that was much debated in Sweden during the last decades of the twentieth century. In the paper, the author, Björn Salomonsson, an experienced child psychiatrist and psychoanalyst, describes his attempts to understand the burdens of the small boy, both at the conscious and unconscious levels. We can follow the psychoanalyst, how he reasons and interprets what he distinguishes following the encounters with the patient. According to Salomonsson (2017) there are very few systematic outcome studies published of psychoanalytical therapy concerning children diagnosed with ADHD. One reason might be that analysts regard the child’s problem to be a sign of neurological disease, which cannot be treated with psychoanalytical or psychodynamic therapy. That is a pity, according to the author of this paper, as it impedes the analysts from learning about the disorder and understanding how these children’s internal worlds are involved in the symptoms they present.

Although some children and young adults suffer from behavioral problems that interfere with their life and future, there are always healthy parts of their personalities that can be encouraged to counterbalance the negative sides. How often do we look at children who are unbalanced from a perspective like that? In his paper, Timimi (2017) presents the Relational Awareness Program (RAP), a clinical non-diagnostic approach toward children, and families, struggling with intense and/or challenging behaviors. Rather than focusing on dysfunctions and symptoms, clinicians working with RAP perceive and approach children and families not as disordered, but as relational and emotional human beings, and prioritize building relationships over controlling behaviors and symptoms. Parents who participated in the RAP sensed that a successful outcome of the interventions was that their “attitude” to their child became more understanding and cooperative. Prior studies indicate that parents of children labeled as having ADHD are encouraged to control their children’s’ behaviors (Erlandsson, Lundin, & Punzi, 2016; Pajo & Stuart, 2012) and thus the RAP approach seems to be a relevant alternative to mainstream interventions that portray the child as dysfunctional.

What happens in the conscious and unconscious mind of a child when the child feels despair or feels that it has been undeserved badly treated? Do we know, or do we have enough imagination to find out? Most children are immensely rich in fantasy and can, if they are allowed, talk about those fantasies and dreams, or can express themselves in drawings or in other artistic forms. Music therapy is one of those therapeutic forms that can be beneficial for children who are hyperactive and have difficulty controlling their emotions. Helle-Valle, Binder, Anderssen, and Stige (2017) have contributed a study concerning music therapy in a Norwegian kindergarten and illustrate how restlessness and hyperactive behaviors might be understood as part of a process rather than as inherent and stable characteristics. They show how children who were described as restless and prone to “fooling around”, during the music therapy project were perceived as cooperative, focused, and creative, thus showing that the current idea of *stable* individual characteristics needs to be challenged. If we approach children and young people with an undifferentiated perception of their beliefs and functioning, it becomes hard to know how to reach out to them. A child, as well as an adult, longs for confirmation, but it is important that the confirmation stems from a reasonably true image of that individual. To give a child who is plagued by inner turmoil an ADHD diagnosis can be perceived as a kind of recognition. However, the problem is that this diagnose is not a confirmation of one specific child—all children receiving the ADHD diagnosis are seen through the same lens. Rojas Navarro and Vrecko (2017) argue in their paper about the effects of ADHD medication in a Chilean school that certain skepticism is warranted regarding analyses suggesting that millions of children in the world should to be seen as fundamentally similar. The researchers present their ethnographic fieldwork, which highlights the complexity and dynamics involved when medicated children work with their teachers and classmates in the classroom setting. The purpose of the study is to demonstrate how the effects of stimulant medication must be seen within the context in which the medication is given.

To summarize, both children and parents might face physical and emotional strain as a consequence of rapid changes in our society, not least in communities where high rates of unemployment lead to economic vulnerability. Parents who lack social networks are in need of recognition and help to maintain their courage and hopes, and parents with an idealized image of family life need support to tone down their expectations. Economic growth and social progress do not guarantee that ethical and humanistic values in our society are preserved and cherished. If they were, things would be different for children and adults who run the risk of being capitalized by a diagnostic culture and the pharmacological industry producing new compounds resulting in increasing profits (Riksrevisionen, 2016). The moral indignation over this state of affairs seems, however, to be rather weak. A hard lesson to learn is that once “the ship” has started to steer a certain route, it takes time to change its course.
